# Elastic, load-bearing and autoclavable protein-based graft for coronary revascularization

**DOI:** 10.3389/fbioe.2025.1732363

**Published:** 2026-01-09

**Authors:** Federica Sallustio, Ikram El Maachi, Dominic Pascal Andre, Alexander Loewen, Amanda Schmidt, Stefan Ruetten, Marius Heitzer, Stefan Jockenhoevel, José Carlos Rodríguez-Cabello, Alicia Fernández-Colino

**Affiliations:** 1 Department of Biohybrid and Medical Textiles (BioTex), AME-Institute of Applied Medical Engineering, Helmholtz Institute, RWTH Aachen University, Aachen, Germany; 2 Electron Microscopy Facility, Uniklinik RWTH Aachen, Aachen, Germany; 3 Department of Oral and Maxillofacial Surgery, Uniklinik RWTH Aachen, Aachen, Germany; 4 Bioforge Lab, LaDIS, University of Valladolid, CIBER-BBN, Valladolid, Spain

**Keywords:** autoclavable, coronary artery bypass, elastin-like recombinamers, miniaturized vascular graft, textile

## Abstract

Autologous grafts, such as the internal mammary artery and saphenous vein, are considered the gold standard for coronary artery bypass. However, there is a critical need for small diameter vascular grafts to meet the demands of coronary artery disease patients, as limitations become especially pronounced due to the extremely small caliber of target vessels. Therefore, we designed and manufactured a miniaturized, autoclavable and synthetic-free vascular graft, composed of elastin-like recombinamers hydrogel and native-like silk fibroin textile to ensure an optimal biological integration and mechanical performance, according to ISO 7198 guideline. The construct demonstrated consistent morphological homogeneity and maintained luminal patency throughout its length. The graft was able to replicate the mechanical performance of the autografts in terms of suture retention and compliance and facilitated the formation of an endothelial monolayer, ensuring a physiologically relevant environment prior to implantation. Moreover, the clinical implantation potential was demonstrated by a successful anastomosis to a human vessel *in vitro*. The proposed graft represents a viable replacement for this clinical application when autografts are not accessible, avoiding a second surgical site and harvesting morbidity.

## 1 Introduction

Coronary arteries provide oxygen supply to the heart itself, maintaining its biological functionality. Any related disease, for instance atherosclerosis, may affect the heart performance and lead to fatal consequences (Shao et al., 2020; Brown et al., 2023). Small diameter vascular grafts (SDVGs) are synthetic or biological conduits adopted to replace or bypass damaged blood vessels with an internal diameter of less than 6 mm (Tanaka et al., 2020).

The current gold standards for coronary artery bypass grafting rely on autologous vessels, precisely internal mammary artery (IMA) and saphenous vein (SV), with IMA demonstrating superior outcomes compared to SV (Suma et al., 1987; Otsuka et al., 2013; McNichols et al., 2021). However, patients with coronary artery disease are more prone to suffer from additional diseases that compromise the integrity of IMA and SV, rendering them as non-ideal for this intervention. Moreover, the use of autologous grafts such as SV is limited by donor site morbidity and the need for an additional surgical field, highlighting the need for off-the-shelf alternatives (Obiweluozor et al., 2020).

In a recent study, Szpytma et al. evaluated the association between vein graft diameter and long-term survival in coronary bypass grafting, and they demonstrated that selecting a vein graft diameter of less than 4 mm can improve long-term survival outcomes (Szpytma et al., 2024). However, synthetic materials widely employed for large diameter vascular grafts, such as Gore-Tex and Dacron (Lang et al., 2024), are not suitable for a small caliber equivalent due to thrombosis (resulting in poor patency) and they exhibit compliance mismatch with the native vessel (Tanaka et al., 2020). Nowadays, the clinical need for SDVG remains unmet, as the challenges associated with vascular grafting are significantly amplified at such reduced diameters.

Natural polymers bioinspired by the native tissues are excellent candidates for vascular tissue engineering, due to their inherent similarities to the biological environment (Moore et al., 2022). Elastin, which accounts for up to 50% of the arterial dry weight, imparts elasticity (e.g., stretching and recoiling) to arterial walls under pulsatile pressure and provides biological support to the endothelial layer lining the lumen (Patel et al., 2006; Wang et al., 2019). Tissue-engineered SDVGs have been fabricated using elastin derived from animal sources (Ryan et al., 2020; Tanaka et al., 2021), but this approach poses limitations in terms of scalability and long-term availability. To overcome these limitations, recombinant technology offers a promising alternative by enabling the controlled, reproducible and safe production of proteins in large quantities through scalable fermentation processes (Mahmoud, 2006; Demain and Vaishnav, 2009). In this context, elastin-like recombinamers (ELRs) play a pivotal role by imitating the pentapeptide present in natural elastin, thereby replicating its inherent elasticity, resilience and biological functionality. In the past years, ELRs exhibited extensive potential in tissue engineering and regenerative medicine (Rodriguez-Cabello et al., 2017; Rodríguez Cabello et al., 2018; Acosta et al., 2020), including those applications that requires contact with blood (González-Pérez et al., 2022).

A SDVG should fulfill both biological and mechanical performance to successfully restore blood flow and increase patient’s life expectancy. Therefore, those requirements should be considered during the design phase. To this end, the incorporation of a well-designed textile, able to function as the load-bearing element, similarly to the circumferentially oriented collagen fibers in the extracellular matrix, constitutes an appealing approach (Singh et al., 2015; Zia et al., 2022; Zhang et al., 2023). Recently, we developed a biohybrid vascular graft composed of a synthetic textile and a macroporous ELRs hydrogel with mechanical properties, including compliance, matching those of native tissue as well as bio- and hemocompatibility, highlighting an *in situ* application potential (Fernández-Colino et al., 2019). Based on these results, Andre et al. fabricated 6 mm-diameter grafts, with an optimized production process and a dedicated preservation scheme to ensure reproducibility, scalability and storage (Andre et al., 2025). However, these studies utilized synthetic textiles (polyvinylidene fluoride and polyethylene terephthalate) in the production of the scaffolds. The use of synthetic materials poses limitations in terms of long-term biocompatibility and remodeling potential compared to natural alternatives (Carrabba and Madeddu, 2018). Moreover, the sterilization phase (e.g., autoclaving), which is essential for clinical translation, must be considered from the early design stages of any vascular graft, as it may affect the material’s structural and biological properties, and thus the safety and performance of the device. A recent study attested the ability of the ELRs to undergo autoclave treatment, without altering or denaturing the core protein structure of the recombinant material (El Maachi et al., 2024).

These factors collectively highlight the need for miniaturized, autoclavable, natural-based grafts tailored for coronary bypass use. Within this study, we aim to develop an autoclavable vascular graft with a diameter of 2 mm, by combining protein-based materials, i.e., ELRs hydrogel and a native-like silk fibroin (NLSF) textile. Achieving such a small diameter, suitable for coronary artery bypass applications, demands precise control over the entire fabrication process. At such miniaturized scale, even minor deviations, such as non-concentric positioning of the textile reinforcement, can compromise the mechanical integrity and uniformity of the graft. The proposed vascular graft is designed to serve as a readily available, suitable alternative for coronary artery bypass procedures.

## 2 Materials and methods

### 2.1 Miniaturized vascular graft fabrication

The elastin-like recombinamers (ELRs) chosen for this study were DRIR-ELR and HRGD6-ELR, both previously described (Flora et al., 2019). In summary, DRIR-ELR is recombinantly engineered for slow degradation due to its low kinetic affinity for the uPa enzyme, while HRGD6-ELR features a cell adhesion-sensitive block. For hydrogel formation via strain promoted azide-alkyne cycloaddition (a catalyst-free click chemistry reaction), the ELRs were chemically modified to include either cyclooctyne or azide groups, as described elsewhere (de Torre et al., 2014).

The warp-knitted textile structure was produced using a MiniTronic 800 double needle bar raschel machine (Rius-Comatex, Spain), with a gauge of E24 (24 needles per inch). A 1 × 1 lapping with a total of 4 yarns (2 yarns on each machine side) was used. The stitch course density was 10 stitch courses per cm. The yarn material used was a native-like silk fibroin (NLSF) multifilament yarn, purchased at Spintex Engineering Ltd. The yarn was fed into the machine by active feed rollers with a yarn run-in of 1750 mm/rack, whereby 1 rack equals 480 stitch courses. Additionally, the textile was thermostabilized in autoclave (DX-Serie Systec, Germany) at 121 °C prior injection molding.

The miniaturized vascular graft (VG) was fabricated exploiting the injection molding technique, in order to achieve an embedded NLSF textile within the ELRs hydrogel matrix (TexELR-VG). The ELR-cyclooctyne (DRIR-ELR) and ELR-azide (HRGD6-ELR) were dissolved at 100 mg/mL in ethanol/PBS 1:1 (v/v) ratio (Sigma Aldrich, United States and Gibco United states, respectively) at room temperature for 30 min. The two ELRs solutions were mixed in a 1:1 volume ratio and injected into a miniaturized, custom-made mold, in which the NLSF textile had been previously positioned, and left at room temperature for 30 min to enable the hydrogel formation, through the click chemistry. ELR-VG tubular scaffolds were fabricated with the same procedure, excluding the incorporation of the NLSF textile.

TexELR-VG and ELR-VG tubular constructs underwent autoclaving as terminal sterilization method for biomedical devices. The samples were autoclaved immersed in PBS at 121 °C and 200 kPa for 15 min (DX-Serie Systec, Germany). The effect of this treatment was evaluated by tensile testing, suture retention, burst pressure, compliance, biological activity and anastomosis.

### 2.2 Structure analysis

We assessed whether the NLSF textile was concentrically located within the ELRs hydrogel, by imaging the samples both cross-sectionally and longitudinally. Specifically, TexELR-VG was left in PBS overnight and imaged with ZEISS LSM 980 with Airyscan 2 confocal microscope (facility IZKF, RWTH Aachen University Hospital, Germany), using a ×10 objective. TexELR-VG was also analyzed by scanning electron microscopy. For that, the samples were first kept in PBS for 2 h and then fixed in 3% glutaraldehyde in Sorensen’s buffer at room temperature for 1 h. After fixation, the samples were subjected to acetone critical point drying. The dried samples were then mounted on aluminum stubs and sputter coated with a 20 nm layer of gold-palladium. Images were captured using The Quattro S microscope (Thermo Fisher Scientific, United States) with an accelerating voltage of 10 kV.

### 2.3 Mechanical properties

Cyclic testing and circumferential tensile testing were performed on ELR-VG, ELR-VG autoclaved, TexELR-VG and TexELR-VG autoclaved, using the Univert uniaxial tensile tester (CellScale Biomaterials Testing, Canada), adhering to ISO 7198:2016 standard (International Organization Standardization, 2016). The sample, in a tubular shape (5 mm long), was immersed in PBS 37 °C for 15 min prior testing. The tests were conducted in PBS at 37 °C. For the cyclic testing, the force-controlled test was carried out for 200 cycles using a load cell of 1 N with a preload of 0.03 N. The applied force was calculated as previously described (Andre et al., 2025). The circumferential tensile testing was performed using 10 N load cell, and applying force until the construct broke. From the resulting stress/strain curve, the breaking stress, Young’s Modulus and maximum strain were calculated. Three replicates were analyzed for each condition and for each test (cyclic and circumferential tensile tests) and the reported values represent mean ± SD across the replicates.

### 2.4 Suture retention

Suture retention was executed on TexELR-VG and TexELR-VG autoclaved, meeting the criteria set by ISO 7198:2016 (International Organization Standardization, 2016). A suture line of 7–0 (Ethicon, United States) was placed 2 mm from the edge of the construct and pulled at a rate of 50 mm/min using a 2.5 N load cell with the Univert uniaxial tensile tester (CellScale Biomaterials Testing, Canada). The test was conducted in a PBS bath at 37 °C. The analysis involved 3 samples per condition.

### 2.5 Burst pressure

Burst pressure was conducted on TexELR-VG and TexELR-VG autoclaved, in accordance with ISO 7198:2016 standards (International Organization Standardization, 2016). A custom chamber fitted with a pressure sensor (JUMO GmbH and Co. KG, Germany) and a syringe pump (Landgraf Laborsysteme HLL GmbH, Germany) was used. The samples, having a tubular shape and a length of 1.5 cm, were left in PBS at 37 °C for 15 min. Subsequently, the scaffolds were sealed on one side to the burst chamber and closed with a stopper on the other side and kept immersed in PBS at 37 °C. The samples were filled with PBS at a rate of 5 mL/min to induce a steady pressure increase until structural failure occurred, indicated by an abrupt pressure drop recorded using LabVIEW (National Instruments, United States). A total of n = 3 for each condition was analyzed.

### 2.6 Compliance

TexELR-VG and TexELR-VG autoclaved scaffolds (n = 3) were assembled in a custom-made bioreactor for compliance testing. The bioreactor consisted of a chamber of polyoxymethylene with transparent polymethylmethacrylate at the sides for optical measurement. The set up was completed with flow and pressure sensors, adjustable resistance and miniaturized centrifugal pump, as reported in Wolf et al. (Wolf et al., 2018) and systematically used in our group (Fernández-Colino et al., 2019; Andre et al., 2025). The compliance device had to be adjusted to hold the scaffold, given its miniaturized diameter. The grafts underwent different pulsatile pressure ranges (50/90, 60/100, 80/120 and 110/150 mmHg) in a bath of PBS at 37 °C, and the diameter and pressure were recorded with a custom-developed LabVIEW program (LabVIEW 7.1; National Instruments). The parameters were extracted through a MATLAB (MathWorks, Natick, Massachusetts, United States) script. Hence, the compliance was calculated as:

$$Compliance=\frac{D_{2}-D_{1}}{D_{1}}\;x\;\frac{1}{p_{2}-p_{1}}\;x\;10^{4}\;\left[\%/100\text{ mmHg}\right]$$

where D_2_ is the maximum diameter measured at the maximum internal pressure P_2_ and D_1_ is the minimum diameter at the minimum internal pressure P_1_.

Furthermore, autoclaved TexELR-VG samples (n = 3) were tested for a long-term compliance at physiological range (80/120 mmHg). The diameter and pressure were recorded every 20 min for 3 h and a mean value of compliance was calculated according to the equation indicated above.

### 2.7 Human umbilical vein endothelial cells (HUVECs) isolation and culture

Human umbilical cords were kindly provided by the RWTH Aachen University Centralized Biomaterial Bank (cBMB) according to its regulations, following RWTH Aachen University, Medical Faculty Ethics Committee approval (cBMB project number 323) after the written consent of the donors at University Hospital Aachen (Germany). HUVECs primary cells were isolated from the umbilical cord, following the protocol described elsewhere (Moreira et al., 2015). HUVECs were cultured on a 2% gelatin (Sigma Aldrich, United States) coated flask in an incubator at 37 °C with 5% CO_2_ and supplied with EGM2 (PromoCell, Germany), supplemented with basic Fibroblast Growth Factor, Insulin-like Growth Factor, Vascular Endothelial Growth Factor 165, Ascorbic Acid, Heparin, Hydrocortisone and FCS (PromoCell, Germany). The medium was changed every 2 days and the cells were used for the experiment in passage three or four.

### 2.8 Evaluation of HUVECs monolayer growth

TexELR-VG autoclaved (2 cm long) was immersed in HUVECs suspension with a concentration of 1 × 10^6^ cells/mL into a 15 mL polypropylene tube, which was then closed with a gas filter to allow CO_2_ exchange. The tube was placed in a sterile environment (37 °C and 5% CO_2_) and rotated along its longitudinal axis at 1 rpm using an RS-TR 05 roller mixer (Carl Roth) for 2 h, to promote cell attachment to the lumen of the graft. After incubation, the autoclaved TexELR-VG was extracted from the cell suspension and transferred to fresh culture medium for static cell culture. The media was changed every 2 days and after 5 days the samples were rinsed with sterile PBS and fixed with 4% paraformaldehyde (Carl Roth, Germany) diluted in PBS at room temperature for 1 h. A total of 3 different donors were tested.

### 2.9 HUVECs staining and confocal visualization

The seeded samples were washed three times with PBS, permeabilized with 0.1% Triton X-100 (Sigma Aldrich, United States) for 5 min and again rinsed three times with PBS. The fixated and permeabilized samples were incubated with 5% Normal Goat Serum (Dako, Denmark) for 1 h at room temperature, followed by 45 min at room temperature and overnight at 4 °C with primary antibody CD31 mouse anti-human (1:100 dilution, Sigma Aldrich, United States) in 1% BSA in PBS, as an endothelial cells’ marker. Afterwards, the samples were washed with PBS three times and incubated for 1 h at room temperature with the secondary antibody goat anti-mouse IgG (H + L) Alexa 568 (1:200 dilution, Invitrogen A11004, United States), followed by three times rinsing in PBS. Later, the nuclei were stained with DAPI solution (Carl Roth, Germany) for 15 min at room temperature and again rinsed three times with PBS. The scaffolds were cut longitudinally and placed on coverslip to image the luminal side with ZEISS LSM 710 confocal microscope (facility IZKF, RWTH Aachen University Hospital, Germany), using a ×20 objective. Images were taken at different parts of the scaffold (top, center and bottom). For CD31 visualization, the samples were excited at 561 nm and the emission was collected at 527–735 nm; whereas, for DAPI visualization the samples were excited at 405 nm and the emission was collected at 410–495 nm. The software Zen black 2012 (Carl Zeiss Microscopy GmbH, Germany) was used for image acquisition. Images acquired from the confocal microscope were used for cell counting, using Image J multi-point tool. Cell density (mean ± SD) was quantified using a fixed area across the three different regions (top, center and bottom) of the scaffold, analyzing samples from three different donors.

### 2.10 Anastomosis

TexELR-VG autoclaved was sutured to a human peritoneal artery (ethical number: EK 219/16) with simple interrupted sutures of 8–0 (Ethicon, United States), resembling the anastomosis in a coronary bypass.

### 2.11 Statistical analysis

All the data were expressed as mean ± SD. The mean ± SD was calculated from n = 3 samples, where each of them was a cut of a specified length from the graft. Each graft corresponds to a single injection-molding fabrication process. Statistical analysis of the mechanical properties and cell density was performed with one-way analysis of variance (ANOVA) with Tukey’s multiple comparison test; whereas, suture retention and burst pressure were analyzed with Welch’s t-test. A *p*-value <0.05 was considered statistically significant. Statistical analyses were performed using GraphPad Prism.

## 3 Results

### 3.1 Fabrication of the miniaturized vascular graft and structural assessment

**FIGURE 1:**
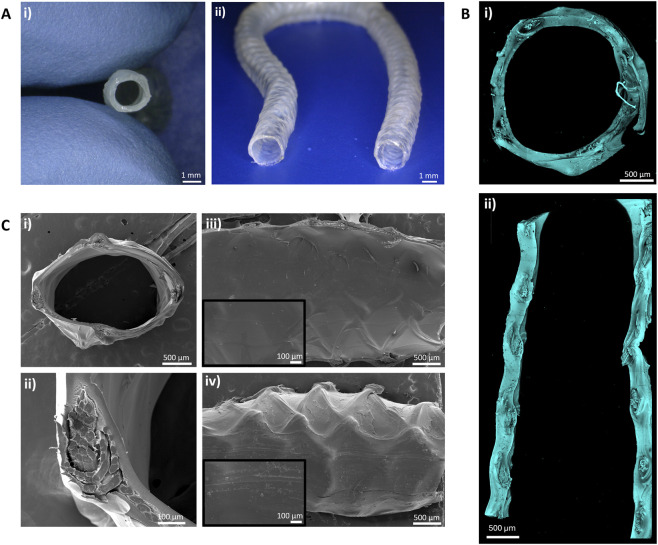
**(A)** TexELR-VG fabricated through injection molding: i) detailed view of the cross section and ii) overall image of a representative graft, where it is apparent its capability to bend without kinking. **(B)** Confocal images of the TexELR-VG: i) cross section and ii) longitudinal view. **(C)** Scanning electron microscopy images of the TexELR-VG: i) cross section, ii) zoom-in of the cross section, where it is visible the NLSF textile embedded in the ELR hydrogel, iii) luminal and iv) abluminal view.

We employed a straightforward and reproducible injection molding technique to fabricate miniaturized and autoclavable vascular grafts (VGs) composed of an ELR hydrogel matrix reinforced with a native-like silk fibroin (NLSF) textile (TexELR-VG). TexELR-VG exhibited a well-defined hollow tubular structure (Figure 1Ai), able to bend without kinking (Figure 1Aii). The crucial stage of embedding the NLSF textile during the injection molding was assessed through confocal and scanning electron microscopy (SEM). The confocal images revealed a concentric positioning of the NLSF textile within the ELR-hydrogel matrix along the cross section and the longitudinal section of the lumen (Figure 1B). This key characteristic was corroborated by SEM analysis of the graft’s cross section, showing a central embedding of the textile. Moreover, the graft presented a smooth surface, in both the luminal and abluminal side (Figure 1C). No signs of degradation or damage were observed after autoclaving (Supplementary Figure S1).

TexELR-VG showed an internal diameter of 1.7 ± 0.2 mm, external diameter of 2.3 ± 0.12 mm and wall thickness of 0.3 ± 0.05 mm. Moreover, we evaluated the effect of terminal sterilization by autoclaving on the dimensions of the graft, which revealed a slight increase in the internal diameter (1.9 ± 0.05 mm), a similar external diameter (2.2 ± 0.09 mm) and a concomitantly smaller wall thickness (0.15 ± 0.04 mm).

**FIGURE 2:**
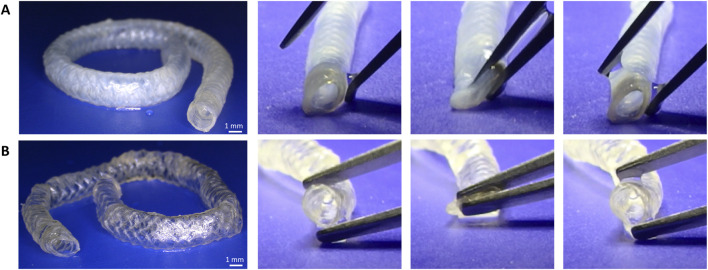
Handleability and flexibility (opening and closing) of **(A)** TexELR-VG and **(B)** TexELR-VG autoclaved performed at 37 °C in PBS.

The graft showed apparent flexibility and kinking resistance (a deformation that can obstruct flow by angulating segments of the graft (Desai and Toole, 1975; Dobrin et al., 2001)) (Figure 2A). This is critically important for ensuring reliable flow through the conduit. Such behavior was present even after terminal sterilization by autoclaving (Figure 2B).

### 3.2 Mechanical properties

**FIGURE 3:**
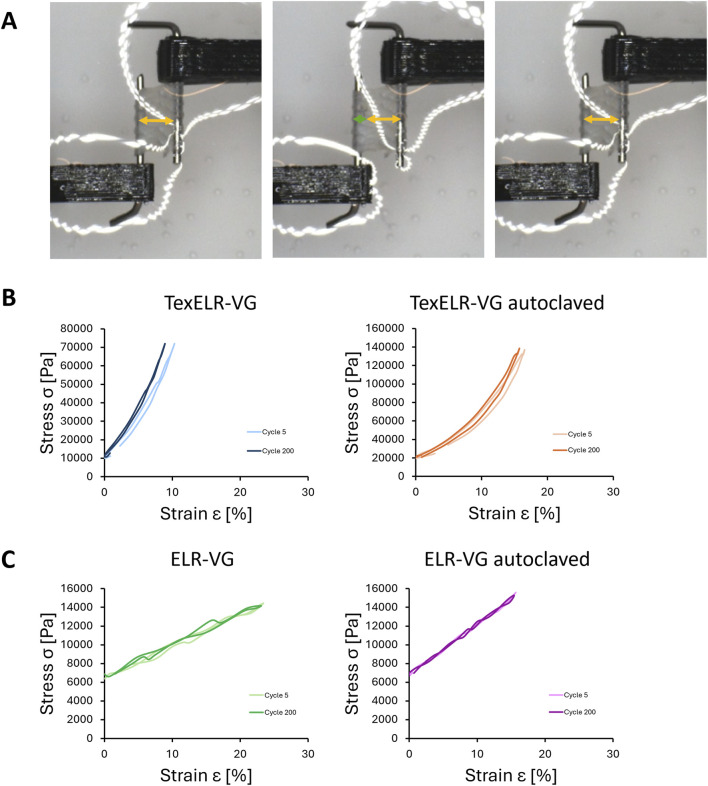
**(A)** Optical images taken during one representative cycle of stretching-recoiling. **(B)** Cyclic testing curves (cycle 5 and 200) of TexELR-VG scaffolds, composed of ELRs hydrogel matrix and NLSF textile. **(C)** Cyclic testing curves (cycle 5 and 200) of ELR-VG scaffolds, composed of pure ELRs hydrogel matrix. The samples (n = 3), having a tubular shape, were analyzed immersed in PBS at 37 °C.

The stress/strain curves of TexELR-VG showed a typical J-shape of natural blood vessel, including coronary arteries (Akentjew et al., 2019), as assessed by cyclic testing (Figure 3A). Such a J-shape persisted after autoclaving. Another common feature of the grafts before and after autoclaving is the presence of hysteresis (Figure 3B). Specifically, TexELR-VG displayed a minor leftward shift in the curve at cycle 200 compared to that at cycle 5. Such a shift was also displayed, but in a lesser extent, by the autoclaved counterpart.

Such hysteresis was not present in the non-reinforced counterparts (i.e., pure ELR-VG and ELR-VG autoclaved), that exhibited a fully linear behavior in the stress/strain curve (Figure 3C), even after 200 cycles of stretching. The perfect elastic behavior of the pure ELR samples points to the presence of the textile as the responsible of the viscoelastic behavior, resulting in dissipation of energy with every cycle (Mavrilas et al., 2002; Stoiber et al., 2020).

**FIGURE 4:**
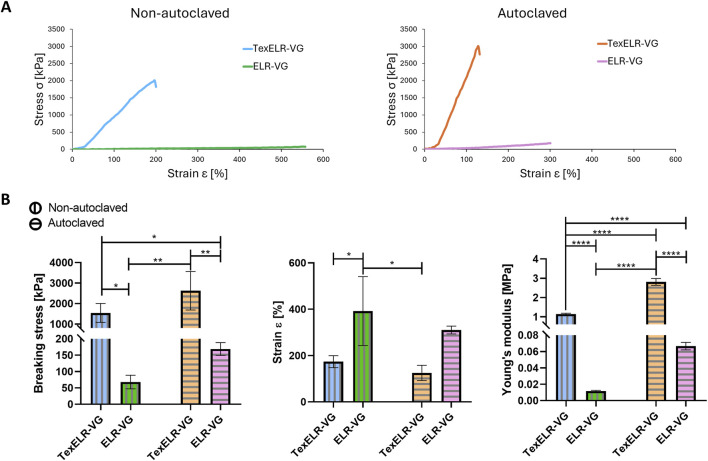
**(A)** Representative stress/strain curves of the circumferential tensile test until rupture of the samples. **(B)** Breaking stress, strain and Young’s modulus of TexELR-VG and ELR-VG grafts. The samples (n = 3), having a tubular shape, were analyzed immersed in PBS at 37 °C. Statistical analysis was performed using one-way ANOVA with Tukey’s multiple comparison test. A threshold of p < 0.05 was used to determine statistical significance (*p < 0.05; **p < 0.01; ***p < 0.001; **p < 0.0001), while p-values greater than 0.05 were considered not statistically significant.

Additionally, circumferential tensile tests were conducted until breaking the samples (Figure 4A). Non-reinforced constructs (i.e., ELR-VG and ELR-VG autoclaved) exhibited breaking stresses of 67.8 ± 17.1 and 168.9 ± 15.9 kPa, Young’s moduli of 11.6 ± 0.7 and 66.6 ± 3.7 kPa, and strain values of 392% ± 112% and 311% ± 14%, respectively. TexELR-VG and TexELR-VG autoclaved showed breaking stress of 1,539 ± 377 and 2,622 ± 767 kPa, and Young’s modulus of 1.1 ± 0.04 and 2.81 ± 0.15 MPa, strain values of 174% ± 21.1% and 125% ± 27.1%, respectively. Therefore, the process of autoclaving did not compromise the mechanical performance of the developed grafts. Indeed, autoclaved grafts showed a trend toward higher Young’s modulus compared to the non-autoclaved counterparts. This difference reached statistical significance in grafts containing textile reinforcement (1.1 ± 0.04 MPa for non-autoclaved vs. 2.81 ± 0.15 MPa for autoclaved TexELR-VG). Notably, the presence of the textile reinforcement statistically increased the breaking stress and Young’s modulus, for both non-autoclaved (breaking stress of 1,539 ± 377 kPa for TexELR-VG vs. 67.8 ± 17.1 kPa for ELR-VG, and Young’s modulus of 1.1 ± 0.04 MPa for TexELR-VG vs. 11.6 ± 0.7 kPa for ELR-VG) and autoclaved grafts (breaking stress of 2,622 ± 767 kPa for TexELR-VG vs. 168.9 ± 15.9 kPa for ELR-VG, and Young’s modulus of 2.81 ± 0.15 MPa for TexELR-VG vs. 66.6 ± 3.7 kPa for ELR-VG) (Figure 4B).

### 3.3 Suture retention and burst pressure

**FIGURE 5:**
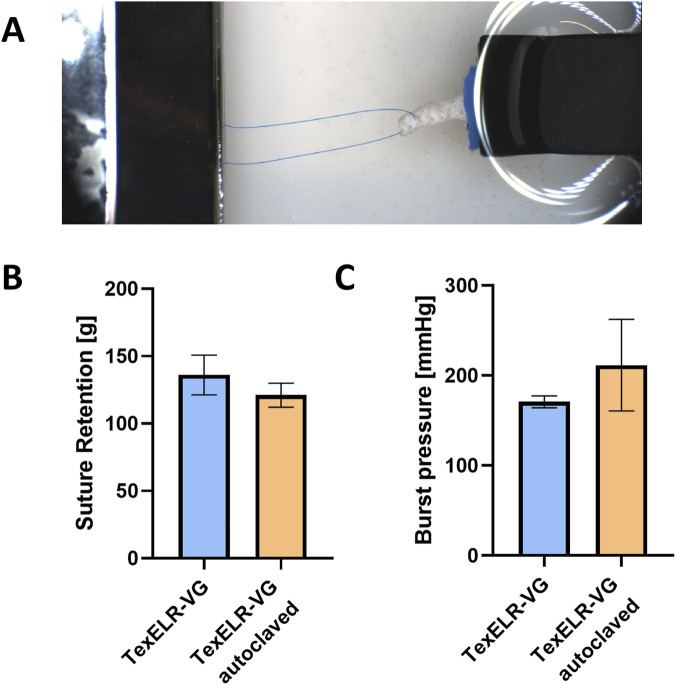
**(A)** Set up for the suture retention. Quantitative analysis of **(B)** suture retention and **(C)** burst pressure for TexELR-VG and TexELR-VG autoclaved. The samples (n = 3), having a tubular shape, were analyzed immersed in PBS at 37 °C. Statistical analysis was performed using Welch’s t-test. A threshold of p < 0.05 was used to determine statistical significance (*p < 0.05; **p < 0.01; ***p < 0.001; **p < 0.0001), while p-values greater than 0.05 were considered not statistically significant.

Suture retention and burst pressure were conducted on our developed graft, following the ISO 7198:2016 guidelines (International Organization Standardization, 2016), before and after autoclaving. Suture retention strength (Figure 5A) of TexELR-VG and TexELR-VG autoclaved was 136 ± 12 and 121 ± 7 g, respectively (Figure 5B). Burst pressure ranged from 171 ± 5.4 mmHg to 211 ± 42 mmHg for TexELR-VG and TexELR-VG autoclaved (Figure 5C).

### 3.4 Compliance

**FIGURE 6:**
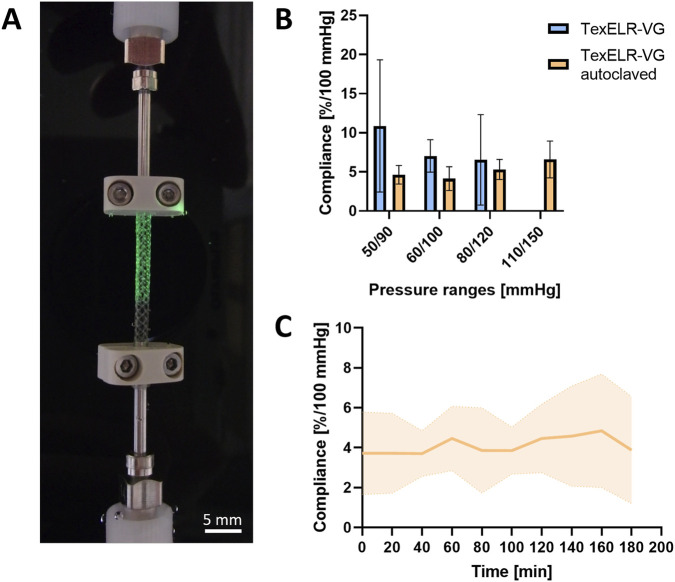
**(A)** Compliance device downscaled to host the miniaturized scaffold. **(B)** Compliance performance of TexELR-VG and TexELR-VG autoclaved at different pressure ranges. **(C)** Compliance of TexELR-VG autoclaved over time. The samples (n = 3), having a tubular shape, were analyzed immersed in PBS at 37 °C.

Compliance performance for both TexELR-VG and TexELR-VG autoclaved (Figure 6A) was examined according to the ISO guideline 7198:2016 (International Organization Standardization, 2016).

TexELR-VG exhibited values of 10.87% ± 6.90%/100 mmHg in the pressure range 50/90 mmHg, 7.04% ± 1.70%/100 mmHg for 60/100 mmHg and 6.55% ± 4.72%/100 mmHg for 80/120 mmHg. This sample was not able to withstand pressures in the range of 110/150 mmHg, due to its complete rupture. TexELR-VG autoclaved showed values of 4.63% ± 0.97%/100 mmHg for 50/90 mmHg, 4.13% ± 1.24%/100 mmHg for 60/100 mmHg, 5.32% ± 1.05%/100 mmHg for 80/120 mmHg and 6.60% ± 1.92%/100 mmHg for 110/150 mmHg (Figure 6B).

Moreover, continuous monitoring of TexELR-VG autoclaved over a 3 h period highlighted the temporal stability of compliance at physiological pulsatile pressure (80/120 mmHg). Specifically, this graft withstood arterial physiological pressure in the long run with a linear trend, revealing an average value of 4.1% ± 1.7%/100 mmHg of compliance over the 3 h (Figure 6C).

### 3.5 Endothelial monolayer formation

**FIGURE 7:**
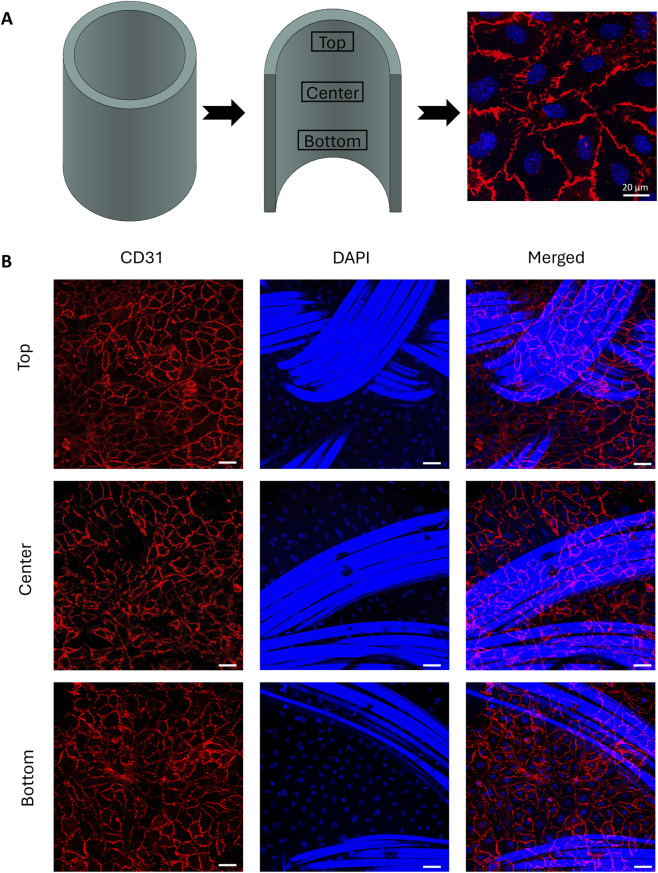
**(A)** Schematic representation of the autoclaved TexELR-VG and representative zoom-in confocal image of the monolayer formed by human endothelial cells. **(B)** Representative images of different regions of the TexELR-VG autoclaved (top, center and bottom) stained for CD31 (red) and DAPI (blue), along with their merged channels. Scale bar 50 µm and magnification × 20.

The attachment of primary human umbilical vein endothelial cells (HUVECs) was assessed in different areas of the scaffold along all its length (Figure 7A). The culture of HUVECs resulted in a complete monolayer covering the lumen of the autoclaved TexELR-VG, supported by the lack of statistically significant variation in cell density among the regions analyzed (Supplementary Figure S2). The marker CD31 was well-expressed throughout all the examined regions of the graft (Figure 7B), confirming an adequate cell-cell interaction (Isacke and Horton, 2000), characteristic of the native endothelium.

### 3.6 Anastomosis to human tissue

**FIGURE 8:**
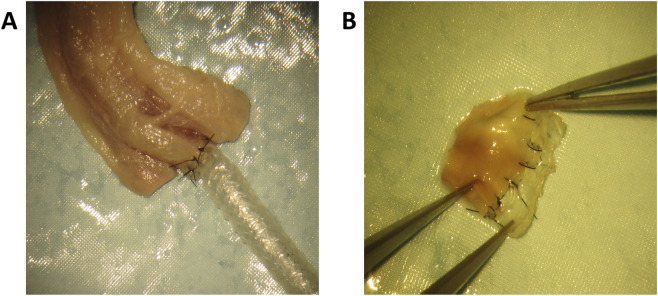
**(A)** Anastomosis of the TexELR-VG autoclaved to the peritoneal artery and **(B)** incised segment of the anastomosed peritoneal artery with good transition between the lumen of the human vessel (left side) and TexELR-VG (right side) with suture material *in situ*.

Anastomosis is a key step during surgery that allows the graft to be attached and connected to the native artery, bypassing the cause of occlusion and permitting the blood to flow again. Herein, we demonstrated the feasibility of suturing the TexELR-VG autoclaved to human peritoneal artery (Figure 8). The graft’s design was compatible with an adequate maneuverability as well as a successful suture to the human vessel. The resulting anastomosis exhibited high resistance to pulling (Supplementary Video S1), suggesting the potential of the graft to be implanted in demanding conditions, as in the coronaries.

## 4 Discussion

Engineered vascular grafts intended for a coronary application need to take into consideration the high-pressure ranges that the blood vessels are subjected to. Such load-bearing capability has to be optimally combined with an elastic behavior, to enable seamless transmission of the pulsatile pressure cycles from the anastomosed vessel to the graft (Hiob et al., 2017). This requires, therefore, the inclusion of seemingly contradictory properties (strength and elasticity). This already poses a challenge from the material point of view, which becomes even more pronounced when dealing with a medical implant that must undergo terminal sterilization.

These frame conditions lead to the rationality of our vascular graft’s design, which entailed a biohybrid approach embedding a load-bearing textile within an elastic matrix. For the fabrication of both components, we selected protein-based materials, avoiding the inclusion of any synthetic components prone to inflammatory or undesirable reactions (Di Francesco et al., 2023). For the load-bearing textile, we exploited Spintex Engineering Ltd. fibers, based on native-like silk fibroin (NLSF), and we created hierarchical structures using warp-knitting. Such textile was embedded in the protein-engineered elastin matrix. These two materials share a key property: they are protein polymers, which are particularly unique given that each has previously been shown individually to withstand terminal sterilization (El Maachi et al., 2024; Schmidt et al., 2025). However, a combination of both materials into a single device, and its subsequent sterilization has not yet been explored.

Our manufacturing approach, based on injection molding in a custom-made mold, enabled us to precisely coat the NLSF textile. Our strategy for ELR-crosslinking exploited the strain-promoted azide-alkyne cycloaddition, characterized by its selectivity, cytocompatibility as well as by its catalyst-free nature (Emerson et al., 2022; Brady et al., 2023). The resulting TexELR-VG graft was characterized by a wall thickness of ∼300 μm, in which the NLSF textile was concentrically located within the ELR hydrogel matrix. The graft featured a smooth surface in both the luminal and abluminal sides, as shown by confocal and SEM analysis (Figures 1B,C). Such a smooth surface has been acknowledged as critically important for blood-contacting devices, to prevent platelet activation and maintain graft patency (Dong et al., 2018; Fernández-Colino et al., 2019; Isella et al., 2025). Additionally, this uniform interface supported endothelialization. Notably, one of the ELR employed in this study incorporates the RGD (Arg-Gly-Asp) motif within its backbone, serving as a bioactive site that promotes integrin-mediated cell adhesion, spreading and proliferation as previously described (Acosta et al., 2020; Hao et al., 2020).

The quasilinear stress/strain graphs obtained for pure ELR-VG underscored the importance of incorporating a textile into the graft’s design to better replicate the physiological behavior of native blood vessels (Loewen et al., 2024). The NLSF textile imparted a non-linear relationship between stress and strain on both non-autoclaved and autoclaved scaffolds (Figure 3B). Specifically, this non-linear relationship followed a characteristic J-shaped curve, which replicates natural blood vessels mechanics. At the onset, small increments in stress lead to significant deformation, allowing the material to stretch with relative ease. However, as the vessel is stretched further, its stiffness increases, requiring much more stress to achieve additional elongation. This behavior prevents overextension and damage while ensuring sufficient blood flow (Shadwick, 1999; Ma et al., 2017; Zhalmuratova et al., 2019). Cyclic tests also showed that TexELR-VG autoclaved reached around 140 kPa of stress at ca. 15% of strain (Figure 3B). This value is in the same order of magnitude of IMA, which is the current gold standard used for coronary revascularization. Specifically, IMA has stress/strain values of approximately 325 kPa at 30% strain (Safar et al., 2000). Importantly, our graft also closely mirrors the stress values of the coronary arteries (100 kPa) (Holzapfel et al., 2005), which are indeed the target location and therefore the vessel they will be connected to. These comparisons should be taken with caution due to the differing testing protocols, but collectively suggest that we closely approach the desired benchmark. No delamination or structural degradation was observed during cyclic testing, confirming robust integration of the NLSF textile within the ELRs matrix. During grafts’ fabrication, the two ELR components are co-injected in their liquid form and subsequently cross-linked via click-chemistry. This fluid processing phase allows for throughout infiltration within the textile structure. The open porosity of the textile further facilitates interlocking with the gelified ELR matrix, thereby enhancing the composite’s overall structural integrity. Future extended cyclic testing and *in vivo* studies will help to confirm long-term stability beyond the time frames tested here. Moreover, the presence of the textile affects the stiffness of the scaffold inducing higher values of breaking stress (Figure 4B). TexELR-VG and TexELR-VG autoclaved exhibited values in the same order of magnitude with respect to the gold standards, i.e., IMA (4,100 kPa) and great SV (2,405 kPa) (Rodríguez-Soto et al., 2022). Moreover, a reduction in strain values for TexELR-VG was observed when compared to non-reinforced counterparts (Figure 4B). The resulting extensibility remained within the range of native vessels, closely matching that of the IMA (134%) and comparable to the SV (242%) (Stekelenburg et al., 2008). Cyclic testing evidenced a full elastic behavior for the scaffolds made of ELR, even after the autoclaving step (Figure 3C). This performance aligns with the intrinsically elastic nature of the ELR, due to their bioinspiration on the amino acid sequence of the natural elastin (Acosta et al., 2020). Regarding the strain before rupture, both ELR-VG and ELR-VG autoclaved (Figure 4B) outperformed the values reached for natural elastin (100%–150%) (Camasão and Mantovani, 2021). This behavior is in accordance with previous finding obtained in our group (El Maachi et al., 2024), highlighting the ability of recombinant technology paired with click-chemistry to surpass the elastic performance of the natural extracellular matrix component. The autoclaving process led to a significant increase in the Young’s modulus of TexELR-VG (Figure 4B), resulting in values slightly higher than that of physiological IMA (1.48 MPa), yet remained below the stiffness typically observed in pathological atherosclerotic coronary arteries (3.77 MPa) (Karimi et al., 2013). Overall, the mechanical enhancement observed post-autoclaving is more likely attributable to supramolecular interactions rather than to molecular-level changes. ELR supramolecular interaction could lead to tighter packing within the polymer chains, leading to enhanced mechanical properties (El Maachi et al., 2024). Autoclaving has also been reported to elicit partial physical crosslinking or aggregation of silk-based materials, in that case by increasing or reorganizing β sheet nanocrystals, which stabilize the network (Qiu et al., 2011). These results suggested the combination of ELR and NLSF textile as a promising approach for a 2 mm vascular graft, given the similarities to IMA and SV.

The burst pressure of a vessel or material is the maximum internal pressure it can endure before rupture. A higher Young’s modulus indicates reduced deformation under load, reflecting greater stiffness and strength. Consequently, materials with higher moduli resist deformation more effectively and typically fail at higher pressures. Autoclaving increased the mechanical properties of the samples, enabling them to withstand higher pressure ranges than the non-autoclaved counterparts. This higher-pressure tolerance directly reflects the mechanical strengthening induced post-autoclaving, likely driven by supramolecular interactions and partial physical crosslinking (El Maachi et al., 2024). The measured burst pressure of TexELR-VG and TexELR-VG autoclaved (Figure 5C) is below that of grafts currently used for coronary bypass (e.g., SV, with values of 1,599 ± 877 mmHg and IMA, with values of 3,196 ± 1,264 mmHg). This indicates the need to adjust the graft design to ensure adequate safety margins for future *in vivo* studies. Specifically, further reinforcement strategies will be explored, focusing on increasing the stitch density of the NLSF textile as well as the concentration of the ELR matrix.

Compliance assessment demonstrated that the TexELR-VG failed to withstand pressures within the 110/150 mmHg range, which is a testing requirement under ISO 7198 guidelines for tubular vascular prostheses (International Organization Standardization, 2016). In contrast, the autoclaved TexELR-VG maintained structural integrity, exhibiting pressure resistance in all the ranges (Figure 6B). Moreover, we performed longer term compliance testing, which showed the capacity of TexELR-VG autoclaved to withstand physiological pressure without bursting (Figure 6C). Its compliance approached the native value for IMA (5.22%/100 mmHg) (Camasão and Mantovani, 2021) and mimicked the one for the great SV (4.40%/100 mmHg) (Montini-Ballarin et al., 2016; Rodríguez-Soto et al., 2022). In this study, compliance was intentionally measured on the autoclaved grafts to directly account for the stiffening induced by the sterilization process, as previously demonstrated by the mechanical properties. Importantly, the autoclaved TexELR-VG exhibited a compliance of approximately 4%/100 mmHg at physiological arterial pressures (80/120 mmHg), which is more than double the values typically reported for clinically used Gore-Tex and Dacron grafts (Tai et al., 2000). Moreover, unlike these synthetic commercial grafts, TexELR-VG is entirely synthetic-free, offering a more biologically favorable environment that can support seamless integration with native tissues.

Any vascular graft intended to restore blood flow needs to be anastomosed. This means that a vascular surgeon attaches the graft to the native vessel, by means of locating suture points, joining the vascular walls and creating a continuous lumen. Therefore, suture retention is a critical parameter for assessing the mechanical integrity of vascular grafts. It ensures that grafts can be safely and effectively implanted, withstand physiological forces and minimize the risk of early failure caused by suture-related complications (Roussis et al., 2015; Guyton et al., 2016). Suture retention tests showed that our developed graft concept exhibited values in accordance with the gold standard currently used for coronary bypass (138 g for IMA) (Konig et al., 2009). In addition, terminal sterilization by autoclaving did not significantly affect the suture retention (Figure 5B), which simplifies clinical workflows and supports regulatory compliance. This aligns with suture retention strength being mainly provided by the NLSF textile in these grafts, which is consistent with previous biohybrid implants where the textile mesh was also the primary contributor to suture holding capacity (Fernández-Colino et al., 2019). Since the NLSF textile is already exposed to 121 °C during its initial thermostabilizing autoclaving step, an additional autoclaving cycle is unlikely to further modify its mechanical properties, resulting in the non-significant change in suture retention.

We also verified the suturability in a closer-to-reality setting. Specifically, the autoclaved graft was successfully sutured by a vascular micro-surgeon to the human peritoneal artery, confirming the absence of leakage and its ability to stretch without tearing (Figure 8; Supplementary Video S1). Through this approach, we establish preliminary evidence supporting the suitability of a 2-mm biobased polymer graft for coronary artery bypass.

Related to its cellular interaction, autoclaved TexELR-VG supported a confluent HUVECs monolayer formation along the full extent of the graft (Figure 7), promoted by the RGD motif in the ELRs employed during the manufacturing (Tugulu et al., 2007; Kumar et al., 2023). The endothelium covers the luminal side of the blood vessels, where endothelial cells function as a dynamic interface between the bloodstream and surrounding tissues, regulating the exchange of nutrients, gases and metabolic waste (Trimm and Red-Horse, 2023). Beyond this barrier function, the endothelium plays a central role in maintaining vascular homeostasis by modulating processes such as coagulation, inflammation and vascular tone. Dysfunction or disruption of the endothelial layer is closely associated with the onset and progression of coronary artery disease and other cardiovascular pathologies (Abrams, 1997; Kinlay et al., 2001; Bockus and Kim, 2022). Moreover, endothelial cells are continuously exposed to shear stress generated by blood flow, a critical biomechanical stimulus that regulates remodeling and the overall vascular homeostasis (Paszkowiak and Dardik, 2003; Katoh, 2023). As reported by Lindner et al. (Lindner et al., 2022), HUVECs are able to sense the shear stress and align in the direction of the flow, reflecting a physiological adaptation characteristic of vascular homeostasis (Roux et al., 2020). Establishing a uniformly confluent HUVEC monolayer provides a foundational platform for subsequent evaluations of shear stress-induced cellular alignment, thereby creating a physiologically relevant model for coronary artery bypass applications. In a previous study, we demonstrated the non-cytotoxic and proliferative environment created by the combination of NLSF fiber and ELR matrix (Sallustio et al., 2025). In this study, we move a step forward by introducing the autoclaving treatment, considered the preferred method for terminal sterilization in clinical practice, as endorsed by the European Medicines Agency (European Medicines Agency, 2019). This approach is especially valued for its consistency, lack of toxicity, and does not generate residual chemicals or by-products.

The promising *in vitro* results open exciting avenues for future investigations, including *in vivo* evaluation to confirm graft integration, long-term functionality and physiological remodeling. Further work on understanding degradation kinetics of the combination of ELRs hydrogel and NLSF textile and performance under dynamic biological conditions will strengthen the clinical potential of this platform. Importantly, both ELRs and silk fibroin have independently demonstrated adequate biocompatibility profiles. Silk fibroin is already established in the clinic, with several silk-based medical devices approved by the FDA (Bucciarelli and Motta, 2022). ELRs, derived from the native elastin pentapeptide sequence, have shown favorable outcomes in animal implantation studies for cardiovascular application (Contessotto et al., 2021). The ELR recombinant sequence allows fine-tuning of degradation kinetics (Flora et al., 2019), providing an effective means to match neo-tissue remodeling. To achieve these optimizations, rigorous *in vivo* studies must be performed, and their results should, if necessary, be fed back into the graft design and ELR selection. Specifically, future *in vivo* studies of these small-diameter vascular grafts, engineered for endogenous remodeling, must comprehensively investigate their biological, mechanical and functional integration within the host environment to secure effective clinical translation and long-term success. Such investigations should analyze the dynamics of host cell interaction, emphasizing the rate, completeness, and spatial uniformity of endothelialization along the graft lumen, as well as assessing its hemocompatibility. Parallel attention must be given to extracellular matrix deposition and remodeling processes, particularly regarding the quality, organization, and biomechanical resilience of the newly synthesized matrix as the scaffold degrades. These efforts will support the advancement of next-generation biobased vascular grafts for coronary bypass applications.

## 5 Conclusion

We have successfully manufactured a vascular graft with 2 mm diameter, characterized by a silk-fibroin textile concentrically placed in the ELRs hydrogel network, to ensure consistent structural and biological features. TexELR-VG autoclaved exhibited mechanical properties (assessed as outlined in the ISO 7198:2016 (International Organization Standardization, 2016)) comparable in terms of suture retention and compliance to the autologous to the autologous grafts employed in coronary artery bypass grafting. The autoclaving treatment not only ensured optimal structural integrity, without hampering the bioactivity, but also rendered the proposed TexELR-VG suitable for off-the-shelf availability for *in vivo* studies. Moreover, HUVECs formed a uniform, confluent monolayer along the lumen of the autoclaved TexELR-VG. These findings underscore the potential of the developed graft as a viable alternative to autologous options, paving the way for further preclinical validation and clinical translation.

## Data Availability

The original contributions presented in the study are included in the article/Supplementary Material, further inquiries can be directed to the corresponding author.
