# Supplementary material for: Elastic, load-bearing and autoclavable protein-based graft for coronary revascularization
Source: Front Bioeng Biotechnol. 2026 Jan 9;13:1732363. doi: 10.3389/fbioe.2025.1732363 (PMC12827764; doi:10.3389/fbioe.2025.1732363)
Supplement: Supplementary file 2 [file DataSheet1.pdf]

Supplementary Information

Elastic, Load-Bearing and Autoclavable Protein-based Graft for Coronary Revascularization

Federica Sallustio[1], Ikram El Maachi[1], Dominic Pascal Andre[1], Alexander Loewen[1], Amanda Schmidt[1], Stefan Ruetten[2], Marius Heitzer[3], Stefan Jockenhoevel[1], José Carlos Rodríguez-Cabello[4], Alicia Fernández-Colino[1*]

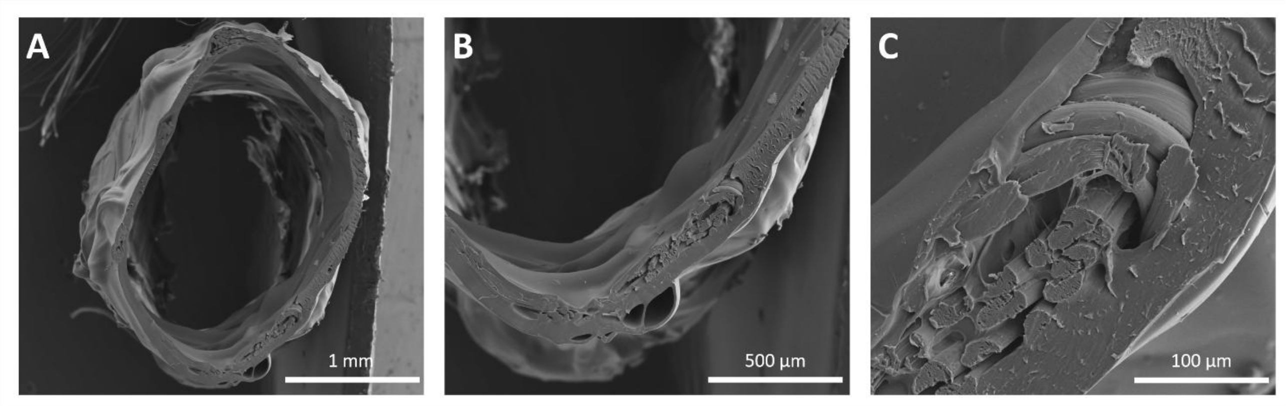

Figure S1: (A) Scanning electron microscopy images of the autoclaved TexELR-VG cross-section, along with (B and C) higher-magnification views.

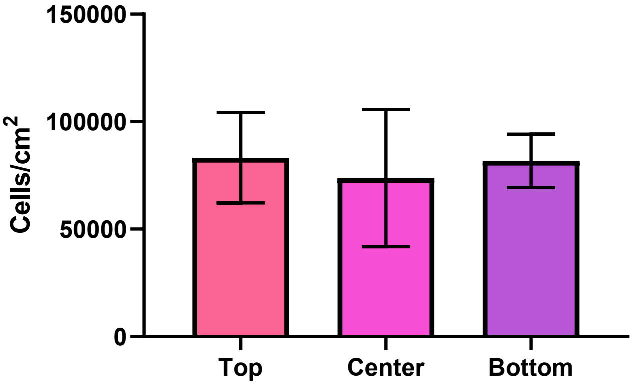

Figure S2: Quantified HUVECs on autoclaved TexELR-VG in the different region of the scaffold. Statistical analysis was performed using One-way ANOVA. A threshold of $p < 0.05$ was used to determine statistical significance (*p < 0.05; **p < 0.01; ***p < 0.001; **p < 0.0001), while p-values greater than 0.05 were considered not statistically significant.

Video 1: Anastomosis of the graft to a human vessel, showing successful maneuverability and high resistance to pulling.
